# Supplementary figures and images for: Molecular features underlying differential SHP1/SHP2 binding of immune checkpoint receptors
Source: eLife. 2021 Nov 4;10:e74276. doi: 10.7554/eLife.74276 (PMC8631942; doi:10.7554/eLife.74276)

Figure 1

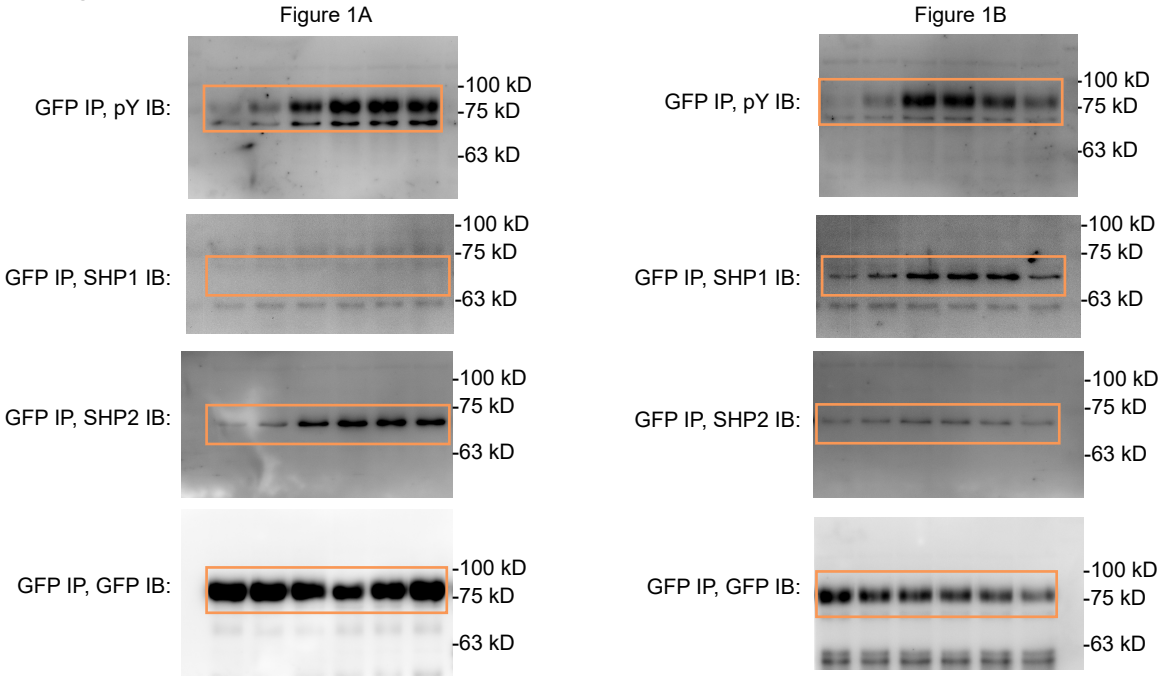

Supplement: Figure 1—source data 1. [file elife-74276-fig1-data1.pdf]

Figure 2A

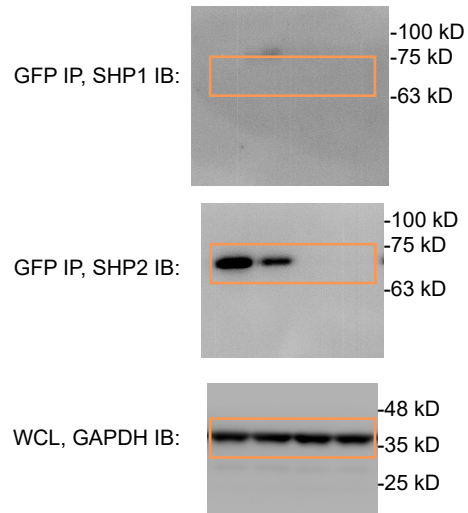

Supplement: Figure 2—source data 1. [file elife-74276-fig2-data1.pdf]

Figure 3-figure supplement 1A

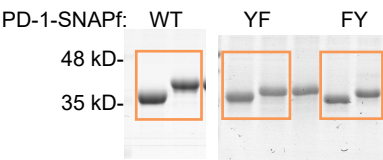

Supplement: Figure 3—source data 1. [file elife-74276-fig3-data1.pdf]

Figure 4C

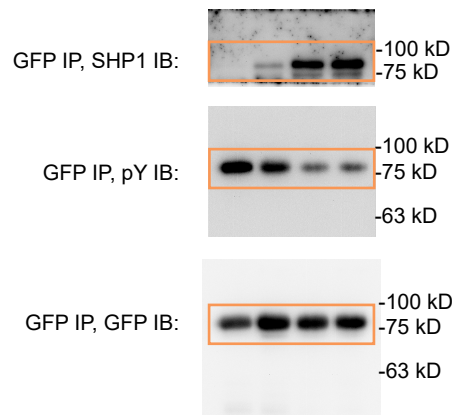

Supplement: Figure 4—source data 1. [file elife-74276-fig4-data1.pdf]

Figure 5B

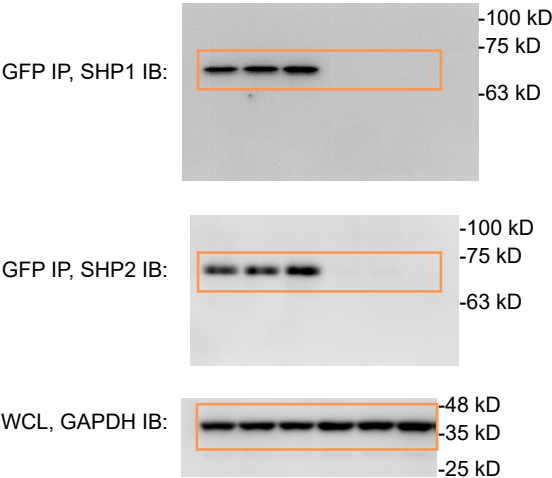

Figure 5-figure supplement 1B

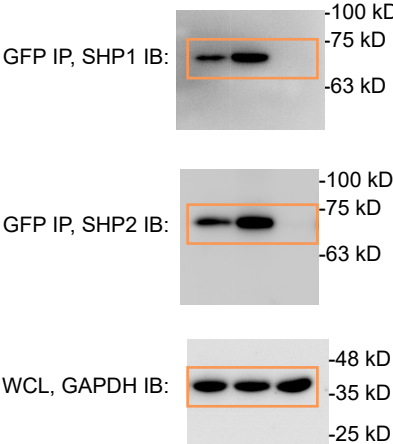

Supplement: Figure 5—source data 1. [file elife-74276-fig5-data1.pdf]

Figure 7B

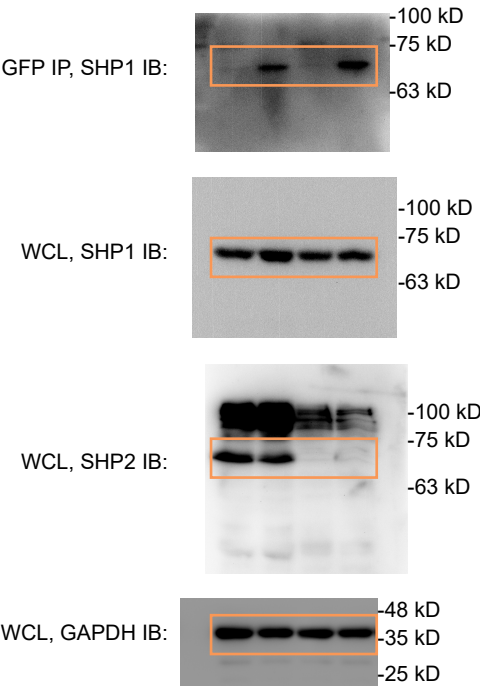

Figure 7-figure supplement 2B

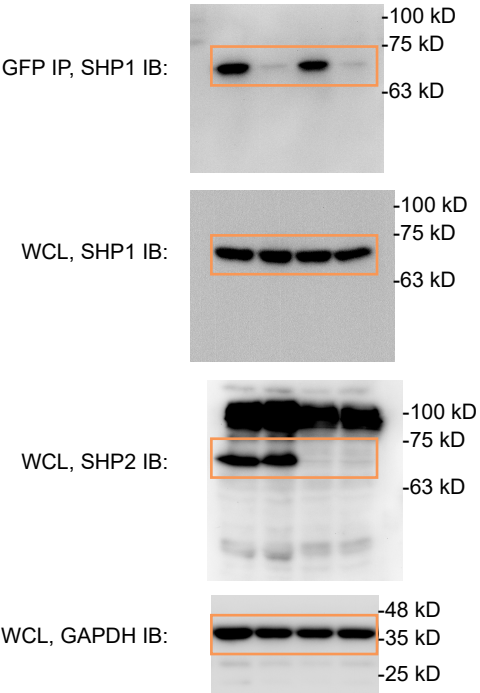

Supplement: Figure 7—source data 1. [file elife-74276-fig7-data1.pdf]

Figure 8

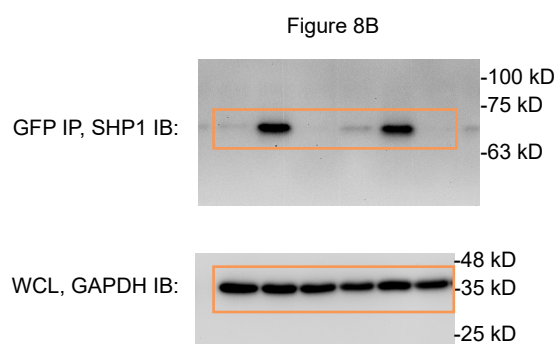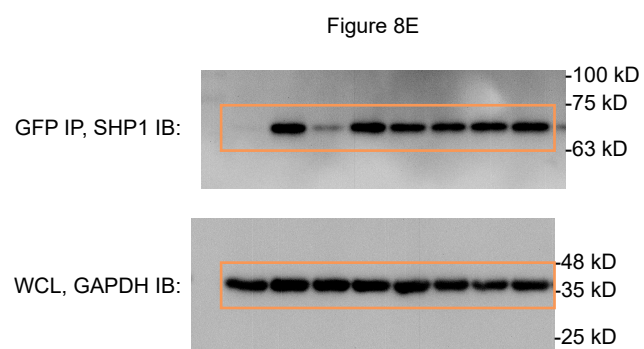

Supplement: Figure 8—source data 1. [file elife-74276-fig8-data1.pdf]
