# Supplementary material for: Molecular features underlying differential SHP1/SHP2 binding of immune checkpoint receptors
Source: eLife. 2021 Nov 4;10:e74276. doi: 10.7554/eLife.74276 (PMC8631942; doi:10.7554/eLife.74276)
Supplement: Supplementary file 1. [file elife-74276-supp1.docx]

**Supplementary File 1.** *K*_d_ values of interactions between individual SH2 of SHP1/SHP2 and phosphorylated ITIM/ITSM of PD-1/BTLA; mean ± s.d. (n = 3).

| *K*_d_ (μM) | PD-1-pITIM | PD-1-pITSM | BTLA-pITIM | BTLA-pITSM |
| --- | --- | --- | --- | --- |
| SHP1-nSH2 | 0.27 ± 0.1 | 0.083 ± 0.020 | 0.064 ± 0.012 | n.d. |
| SHP1-cSH2 | n.d. | 1.7 ± 1.4 | 1.8 ± 0.20 | 0.86 ± 0.31 |
| SHP2-nSH2 | 0.38 ± 0.10 | 0.14 ± 0.049 | 0.34 ± 0.082 | 2.1 ± 1.1 |
| SHP2-cSH2 | 1.4 ± 0.57 | 0.10 ± 0.029 | 1.1 ± 0.54 | 1.1 ± 0.19 |

n.d.: not detected
