# Supplementary material for: Molecular features underlying differential SHP1/SHP2 binding of immune checkpoint receptors
Source: eLife. 2021 Nov 4;10:e74276. doi: 10.7554/eLife.74276 (PMC8631942; doi:10.7554/eLife.74276)
Supplement: Supplementary file 2. [file elife-74276-supp2.docx]

**Supplementary File 2.** Table summarizing ∆G of PD-1/BTLA:SHP1/SHP2-tSH2 interactions in a parallel or an anti-parallel mode.

| ∆G (kJ/mol)^a^ | Parallel | Anti-Parallel |
| --- | --- | --- |
| PD-1:SHP1-tSH2 | -70.4 | -40.4 |
| PD-1:SHP2-tSH2 | -76.6 | -72.5 |
| BTLA:SHP1-tSH2 | -75.7 | -32.8 |
| BTLA:SHP2-tSH2 | -70.9 | -66.4 |

a: ∆G = RT*ln (K_d_), showing the change of Gibbs free energy;

T = 298.15 K, R is molar gas constant, R = 8.314 × 10^-3^ kJ/K/M
