# Supplementary material for: Molecular features underlying differential SHP1/SHP2 binding of immune checkpoint receptors
Source: eLife. 2021 Nov 4;10:e74276. doi: 10.7554/eLife.74276 (PMC8631942; doi:10.7554/eLife.74276)
Supplement: Supplementary file 3. [file elife-74276-supp3.docx]

**Supplementary File 3.** Table summarizing ∆G of individual SH2:ITIM/ITSM interactions

| ∆G (kJ/mol)^a^ | SHP1-nSH2 | SHP1-cSH2 | SHP2-nSH2 | SHP2-cSH2 |
| --- | --- | --- | --- | --- |
| PD-1-pITIM | -37.5 | 0.0 | -36.6 | -33.4 |
| PD-1-pITSM | -40.4 | -32.9 | -39.1 | -40.0 |
| BTLA-pITIM | -41.1 | -32.8 | -36.9 | -34.0 |
| BTLA-pITSM | 0.0 | -34.6 | -32.4 | -34.0 |

a: ∆G = RT*ln (K_d_), showing the change of Gibbs free energy;

T = 298.15 K, R is molar gas constant, R = 8.314 × 10^-3^ kJ/K/M
