# Supplementary material for: Molecular features underlying differential SHP1/SHP2 binding of immune checkpoint receptors
Source: eLife. 2021 Nov 4;10:e74276. doi: 10.7554/eLife.74276 (PMC8631942; doi:10.7554/eLife.74276)
Supplement: Supplementary file 4. [file elife-74276-supp4.docx]

**Supplementary File 4.** A list of immunoreceptors that contain both ITIM and ITSM

| Receptor | ITIM Y position  (AA) | ITSM Y position  (AA) | ITIM-ITSM distance (AA)^a^ |
| --- | --- | --- | --- |
| PD-1 | 223 | 248 | 25 |
| BTLA | 257 | 282 | 25 |
| Siglec-3 | 340 | 358 | 18 |
| Siglec-5 | 520 | 544 | 24 |
| Siglec-6 | 426 | 446 | 20 |
| Siglec-9 | 433 | 456 | 23 |
| Siglec-11 | 644 | 668 | 24 |
| CD300LF | 249 | 284 | 35 |
| VSTM4 | 289 | 310 | 21 |
| SIRPα | 429  470  496 | 453 | 24  -16  -43 |
| SLAMF5 | 341 | 279  316 | -62  -25 |
| SLAMF6 | 274 | 285  309 | 11  35 |
| PECAM1 | 690 | 713 | 23 |
| MPIG6B | 211 | 237 | 26 |
| AA: amino acids  ITIM: immunoreceptor tyrosine-based inhibition motif  ITSM: immunoreceptor tyrosine-based switch motif  a: Calculated as (ITSM Y position) - (ITIM Y position) | | | |
